# Tissue Depletion of Taurine Accelerates Skeletal Muscle Senescence and Leads to Early Death in Mice

**DOI:** 10.1371/journal.pone.0107409

**Published:** 2014-09-17

**Authors:** Takashi Ito, Natsumi Yoshikawa, Takaaki Inui, Natsuko Miyazaki, Stephen W. Schaffer, Junichi Azuma

**Affiliations:** 1 College of Pharmacy, Department of Clinical Pharmacogenomics, Hyogo University of Health Sciences, Kobe, Hyogo, Japan; 2 College of Medicine, Department of Pharmacology, University of South Alabama, Mobile, Alabama, United States of America; University of Florida, United States of America

## Abstract

Taurine (2-aminoethanesulfonic acid) is found in milimolar concentrations in mammalian tissues. One of its main functions is osmoregulation; however, it also exhibits cytoprotective activity by diminishing injury caused by stress and disease. Taurine depletion is associated with several defects, many of which are found in the aging animal, suggesting that taurine might exert anti-aging actions. Therefore, in the present study, we examined the hypothesis that taurine depletion accelerates aging by reducing longevity and accelerating aging-associated tissue damage. Tissue taurine depletion in taurine transporter knockout (TauTKO) mouse was found to shorten lifespan and accelerate skeletal muscle histological and functional defects, including an increase in central nuclei containing myotubes, a reduction in mitochondrial complex 1 activity and an induction in an aging biomarker, Cyclin-dependent kinase 4 inhibitor A (p16INK4a). Tissue taurine depletion also enhances unfolded protein response (UPR), which may be associated with an improvement in protein folding by taurine. Our data reveal that tissue taurine depletion affects longevity and cellular senescence; an effect possibly linked to a disturbance in protein folding.

## Introduction

Senescence is one of the major contributors to the development and progression of diseases, suggesting that regulation of senescence could significantly benefit general health. To date, diet restriction is the most reliable means of slowing the aging process [1]. Hence, dietary intervention could represent a strategy for regulating aging. Indeed, several nutrients and drugs, such as resveratrol [2], polyamine [3], aspirin [4] and rapamycin [5], [6], appear to extend lifespan.

Taurine (2-aminoethanesulfonic acid) is a candidate for use against aging. Taurine is widely distributed in vertebrate animals, whose tissue taurine concentration averages 1–20 micromol/g tissue. These large levels of taurine are maintained through both the diet and hepatic biosynthesis in human beings. The main source of dietary taurine is meat, with seafood being particularly rich in taurine [7]. For some individuals, energy drinks are another source of taurine. A multi-country epidemiological study revealed that dietary taurine intake is negatively correlated with mortality from ischemic heart disease [8]. Pharmacological and nutritional administration of taurine has been shown effective against a wide variety of diseases in both animal and human studies [9]–[14]. On the other hand, dietary taurine insufficiency in species with low rates of taurine biosynthesis, such as cats and foxes, leads to several disorders, including retinal degradation, dilated cardiomyopathy and reproductive defects [15]–[17].

Taurine, a zwitter ion, possesses many biological actions, one of the important ones being its role as an organic osmolyte [18]. Besides regulating cell volume and osmolality, organic osmolytes act as chemical chaperones to modulate protein folding in the endoplasmic reticulum (ER) [19]. For instance, taurine as well as the other organic osmolytes, promote proper protein folding and membrane trafficking of the mutant cystic fibrosis transmembrane conductance regulator (CFTR) protein (delta508 CFTR) which fails to move out of the ER and into the plasma membrane without an organic osmolyte [20]. In addition, taurine modulates calcium transport [21], suppresses the generation of reactive oxygen species [22] and inhibits cellular apoptosis [23]. All of these actions are thought to contribute to the maintenance of cellular homeostasis. At the same time they likely retard the aging process. However, there is little evidence showing that taurine exhibits anti-aging activity.

In this study, we evaluated the role of taurine on longevity and tissue aging using a tissue taurine deficient mouse model generated by knocking out the taurine transporter (TauT) [24]. We found that tissue taurine depletion shortens the lifespan of mice and accelerates skeletal muscle aging. We further demonstrate that tissue taurine depletion initiates ER stress and stimulates unfolding protein response (UPR) of taurine-depleted muscle. Our findings suggest a novel role of taurine on tissue aging which may be, at least in part, associated with chaperone-like activity of taurine.

## Materials and Methods

### Ethics statement

Animal studies were carried out in accordance with an animal protocol approved by the Institutional Animal Care and Use Committee of Hyogo University of Health Sciences. *In vivo* wound healing assay was performed under anesthesia with 2% isoflurane inhalation. Mice were euthanized by CO_2_ inhalation or cervical dislocation, and all efforts were made to minimize suffering of the animals.

### Animal experiment

TauTKO and littermate mice (C57BL/6 background) were obtained by breeding heterozygous male and female. Mice were housed in Specific pathogen-free (SPF) environment, fed a standard chow (MF, Oriental Yeast, Japan), had access to water *ad libitum* and maintained on a 12-h light/dark cycle. Mice (total number; n = 37 (wild-type (WT)), n = 20 (heterozygous (hetero)) and n = 58 (TauTKO)) were monitored for survival once per one or two days by our veterinary staff. During monitoring survival, apparent sick and injury were not observed. Young (3-month-old, TauTKO n = 3, wild-type n = 3) and old (17 to 22-month-old, n = 17 (TauTKO), n = 10 (hetero), n = 16 (WT)) mice were euthanized to isolate tissues for each experiments.

### RNA isolation and real-time PCR

Total RNA was isolated from skeletal muscles of TauTKO and WT mice by using Sepazol (Nacalai tesque, Japan), and cDNA was generated from total RNA by the reverse transcription with Rever Tra Ace (Toyobo, Japan). Quantitative RT-PCR analyses were performed by using Applied Biosystems Step One Plus (Applied Biosystems) with THUNDERBIRD SYBR qPCR Mix (Toyobo, Japan). The primers used are shown in Table S1. GAPDH was used as an internal control.

### Histological analysis

Sections from frozen tissues were cut by cryostat (Carl Zeiss). Sections we stained by Hematoxylin&Eosin method [24].

### Mitochondria complex

Mitochondria were isolated from skeletal muscle as described elsewhere with slight modifications [25]. Hindlimb muscles were finely minced and were incubated with trypsin (0.1 mg/mL) in mitochondria isolation medium (100 mM sucrose, 10 mM EDTA, 46 mM KCl, 100 mM Tris-HCl, pH7.4) at 4°C for 10 min. Digestion was stopped by adding fatty acid-free BSA (1 mg/mL) and trypsin inhibitor (0.65 mg/mL), and then tissues were homogenized by potter homogenizer. The homogenate was centrifuged at 600×g 10 min and the resulting supernatant was centrifuged again at 8500×g for 15 min. Supernatant and the fluffy upper layer were discarded and the mitochondrial pellet was suspended in isolation medium. After sonication, mitochondria lysate was stored at −80°C. Protein concentration in mitochondria lysate was assayed by bicinchoninic acid assay. Electron transport chain complexes were assayed with isolated mitochondria as described previously [26].

### Western blots

Tissues were homogenized in RIPA buffer (10 mM Tris-HCl, pH8.0, 150 mM NaCl, 1 mM EDTA, 0.5% nonidet-40, 0.5% deoxycholate, 0.5% SDS, protease inhibitor cocktail, phosphatase inhibitor cocktail (Nacalai tesque) by Potter homogenizer and then sonicated on ice for 15 s by using a microtip sonicator. After centrifusion, protein concentration of supernatant was determined by bicinchoninic acid assay (Nacalai tesque). SDS sample buffer (x2∶125 mM Tris-HCl, pH 6.8, 4% SDS, 10% mercaptoethanol, 20% Glycerol, 0.002% bromophenol blue) was added into lysates. The membrane transfer was performed by semi-dry method. Western blot was performed as described previously [27]. Anti-Grp78 (BD bioscience), XBP1 (Abcam), GAPDH (Milipore) antibodies were used.

### Microarray and pathway analysis

RNA from skeletal muscle was isolated by using Sepazol-RNA super G, and cleaned by using RNeasy mini kit (Qiagen). A microarray analysis was performed by using SurePrint G3 Mouse Gene Expression 8×60K arrays (Agilent Technologies) according to the manufacturer’s instructions. The microarray data have been deposited in Gene Expression Omnibus (GSE57373). Data analysis was performed with GeneSpring software (version GX 10.3). All comparisons of expression levels between the groups were performed using un-paired t-tests. Genes were identified as differentially expressed if they showed a fold-change of at least 1.8 with a p value lower than 0.05. Pathway analysis was performed by using Ingenuity Pathway Analysis (IPA) software (Agilent technology). The Fisher’s exact test was used to determine the statistical significance of association with the recorded knowledge concerning molecular networks, upstream regulators and biological functions in IPA.

### Statistics

Student’s t-test or Tukey-Kramer test (for multiple comparisons) was used to determine statistical significance between groups. Log lank test was used in the longevity study. Differences were considered statistically significant when the calculated P value was less than 0.05.

## Results

Knocking out TauT causes a 99% decrease in taurine content in muscle and heart and ∼90% decline in brain, adipose tissue and liver as well as about 75% decline in plasma taurine concentration [24], [28]. TauTKO mice exhibit a variety of typical phenotypes, such as lower body weight, exercise intolerance and muscle atrophy [24], [29]. Moreover, various disorders, such as a mild cardiomyopathy [24], blindness [28] and liver fibrosis [30], commonly develop with advanced aging, indicating that tissue taurine deficiency may relate to senescence. However, the impact of tissue taurine depletion on aging and longevity has not been elucidated in the TauTKO mouse model.

### Shortened longevity in TauTKO mice

**Figure 1:**
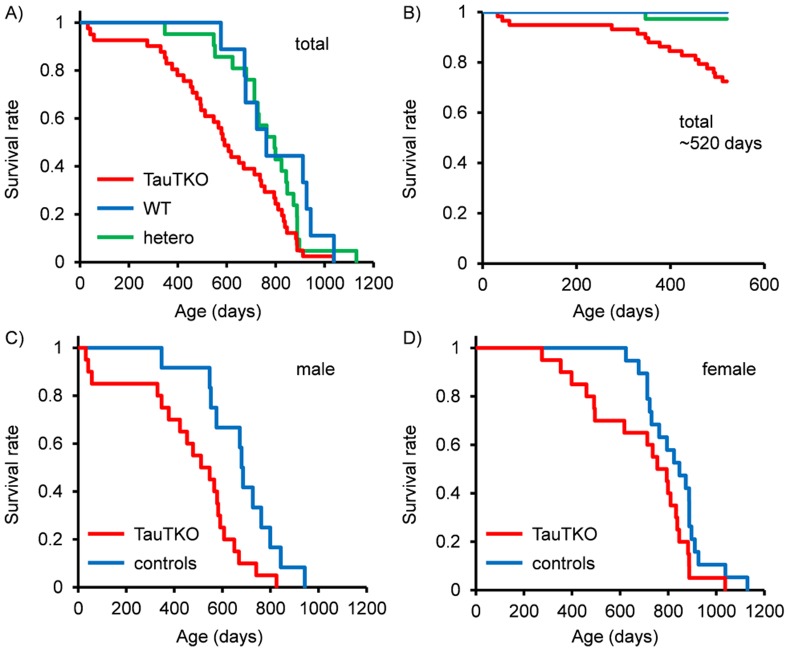
Shortened lifespan of TauTKO mice. Kaplan-Meier survival curves for (A,B) combined (C) male and (D) female wild-type (WT) heterozygous (hetero) and TauTKO mice. (A) n = 21 (WT), 10 (hetero), 41 (TauTKO) (B) n = 37 (WT), 20 (hetero), 58 (TauTKO). (C) n = 12 (control; WT+hetero), 20 (TauTKO) (D) n = 19 (conrol; WT+hetero), 21 (TauTKO). Data for B), but not A), C) and D), includes the mice sacrificed over 520 days after birth.

The lifespan of TauTKO mice and their littermate controls that were used solely for the lifespan experiment was monitored (total number; n = 21 (wild-type (WT)), n = 10 (heterozygous (hetero)) and n = 41 (TauTKO). Log-rank test analysis showed a significant difference in the survival curves of TauTKO, hetero and wild-type mice. The median life span of the mice varied from 795 days for WT, 723 days for hetero and 591 days for TauTKO mice (χ^2^ = 7.83, *P* = 0.020, Fig. 1A). Some mice older than 520 days (about 1.5 years old) of age that were not included in the lifespan analysis were used in another experiment. To prevent the introduction of an artifact we analyzed survival rate until 520 days including the mice sacrificed over 520 days after birth (total number; n = 37 (WT), n = 20 (hetero) and n = 58 (KO)). Those experiments also showed significant differences in the survival rate of TauTKO, hetero and WT mice (χ^2^ = 5.99, *P* = 0.00054, Fig. 1B). Analysis of each sex separately showed that median lifespan of the male mice varied from 686 days in the WT+hetero group and 511 days for the TauTKO group (χ^2^ = 6.11, *P* = 0.013, Fig. 1C). In contrast, deletion of *TauT* in female mice had little effect (median survival: 847 days (WT+hetero) and 794 days (TauTKO)) (χ^2^ = 2.89, *P* = 0.089, Fig. 1D).

### Induction in Cyclin-dependent kinase 4 inhibitor A (p16INK4a) in TauTKO tissues

**Figure 2:**
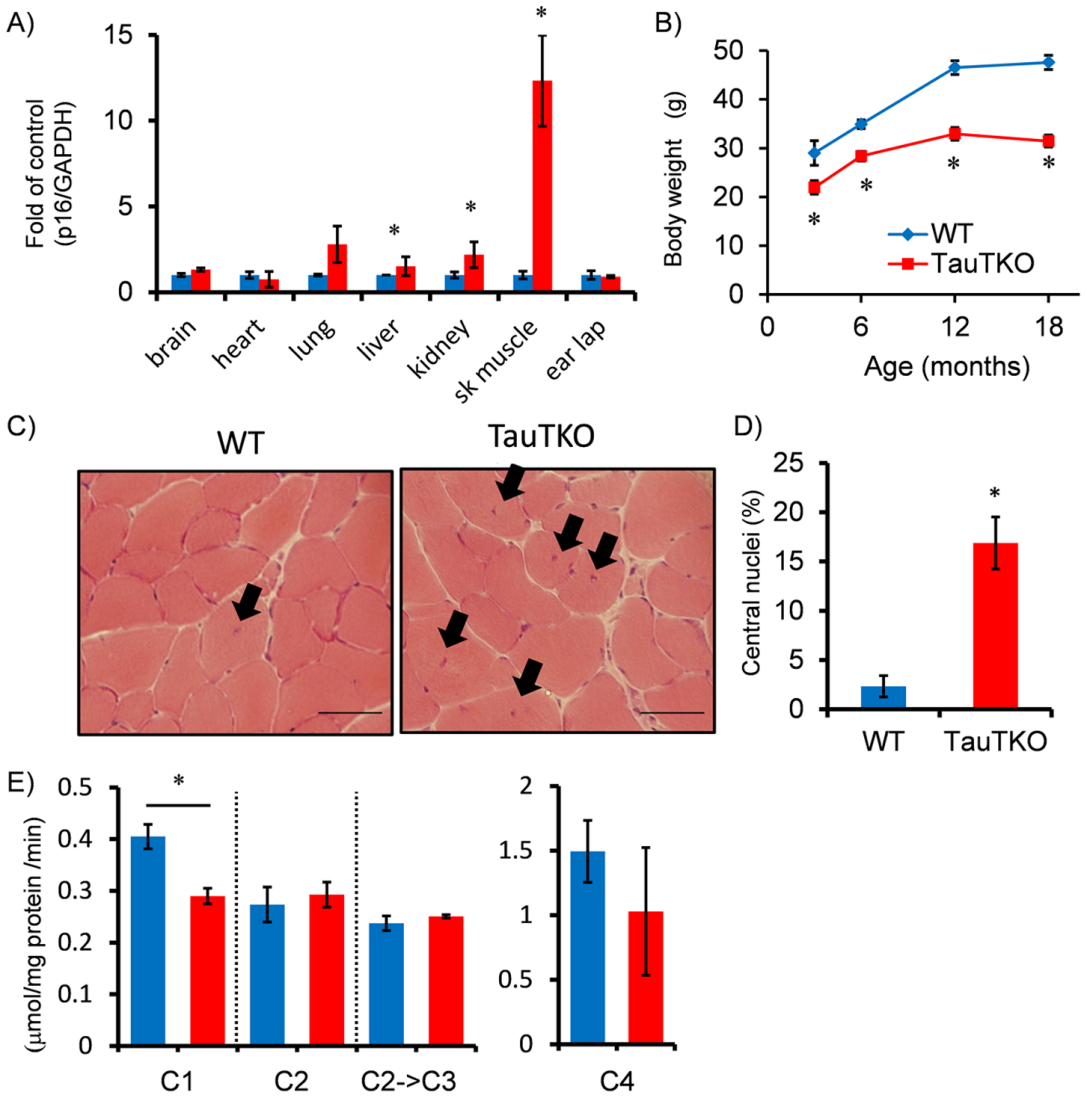
Accelerated aging in TauTKO muscle. A) mRNA level of the senescence marker, p16INK4A, was quantified in several tissues of old TauTKO and WT mice by real-time RT-PCR. The marker is severely increased in old TauTKO muscle. n = 4–7. B) Body weight of TauTKO mouse is consistently lower than that of the WT mouse. n = 6–12. C), D) Histological analysis of tibial anterior muscles shows an induction of myotubes with central nuclei indicated by arrows in old TauTKO muscle. The ratio of myotubes with central nuclei to total myotubes is significantly higher in old TauTKO than WT. n = 6. Scale bar = 50 µm. E) The assays for electron transport chain complex reveal that activity of mitochondrial complex1 is decreased in old TauTKO muscle. C1–4; Complex 1–4. n = 4–5. *;p<0.05.

To determine whether tissue taurine depletion enhances tissue senescence, we first evaluated in tissue of aged (18 to 22-mo) mice the expression of p16INK4a, which is a robust biomarker of cellular aging [31]. The most pronounced increase in p16INK4a content was observed in skeletal muscle of aged male TauTKO mice, with levels increasing more than 10 times those of aged WT mice (Fig. 2A). Inductions of p16INK4a mRNA were also observed in lung and kidney of aged TauTKO mice although the level was significantly less than that seen in muscle. The level of p16INK4a mRNA was not changed in earlap (skin), heart and liver between TauTKO and WT mice. Similarly, p16INK4a was induced more in aged female TauTKO compared to aged female WT mouse although the increase in levels was not as severe (Figure A in Fig. S1). Meanwhile, the expression of p16INK4a in young TauTKO and young WT was not different (Data not shown), suggesting that its induction in old TauTKO is due to tissue aging.

### Aging-related phenotype in skeletal muscles of TauTKO mice

One of the features of skeletal muscle aging is sarcopenia, which is characterized by a decrease in the size of the muscle fibers. Yet, muscle size of young TauTKO mice is already reduced and associated with consistently lower body weight (Fig. 2B) and diminished exercise tolerance capacity [24]. Nonetheless, upon aging (>18-months of age) many fibers of TauTKO muscle, but not those of WT mice, undergo changes in the distribution of the nuclei, which become centralized in TauTKO fiber (Fig. 2C). Central nuclei are a feature of regenerating muscle fiber, observed not only in dystrophic muscle but also in aged muscle [32], [33]. However, the number of fibers with central nuclei was significantly higher in older TauTKO than older WT mice, a feature not observed in younger TauTKO mice (Fig. 2D). While aged TauTKO female muscle also exhibits a significant increase in myotubes containing central nuclei (Figure B in Fig. S1), the difference between WT and TauTKO muscle relative to the number of centrally localized nuclei is less in females than in males.

Another characteristic feature of aging muscle is the decline in respiratory chain complex I activity [34]. Activity of complex 1 was suppressed in muscle mitochondria of aged TauTKO mice (Fig. 2E), while the activity of the other complexes were not significantly different in mitochondria isolated from aged TauTKO and aged WT muscle. Meanwhile, mitochondrial complex 1 activity as well as other complex activities was not different between young WT and TauTKO muscles (Data not shown).

Moreover, we analyzed red-ragged fibers, which are markers of abnormal subsarcolemmal aggregates of mitochondria characteristically present in one of the mitochondrial diseases [35], since it has been suggested that the lack of TauT might cause a mitochondrial encephalopathy-related phenotype [36]. However, in TauTKO muscle we failed to detect an increase in red-ragged fibers (Figure C in Fig. S1).

### Microarray analysis

**Figure 3:**
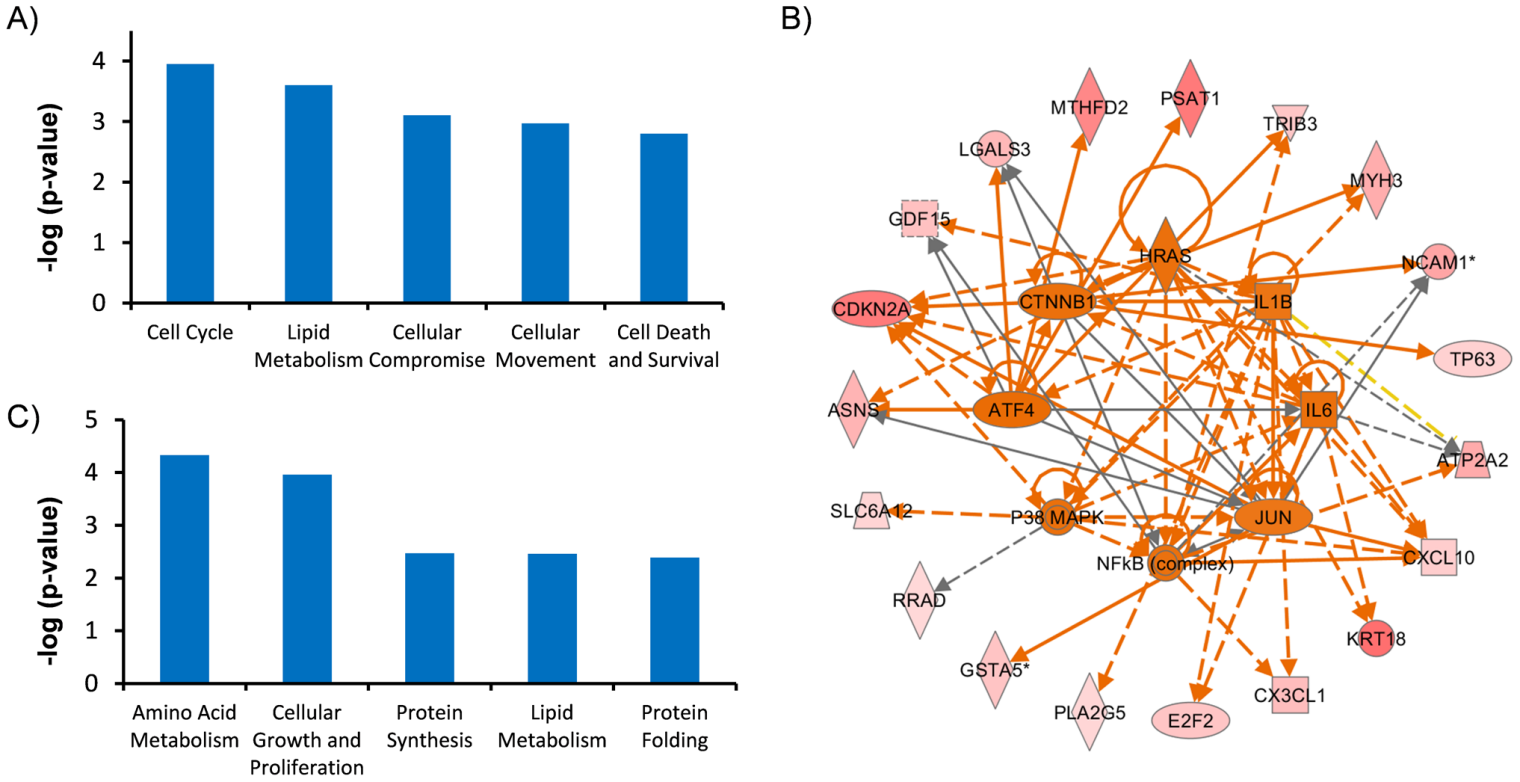
Specific changes in aged and young TauTKO skeletal muscle. A) Biological functions analysis shows a significant activation of cell cycle progression, prostaglandin D2 release, degranulation of cells, cell movement and cell death (necrosis, apoptosis) in aged TauTKO muscle. B) Wheel network diagram showing the overlap of the major upstream regulators of genes chaged in old TauTKO muscles. Each association between genes and upstream regulators is listed in Table 1. Red shapes: upregulated gene, green shapes: downregulated gene, orange shapes: predicted activation, Orange arrows: leads to activation, yellow arrow: findings inconsistent with prediction, gray arrows: effect not predicted. C) Biological functions analysis shows a significant activation of neural amino acid transport, colony formation of cells, protein synthesis, protein folding and steroid content both in young and aged TauTKO muscle compared to age-matched control littermate.

**Figure 4:**
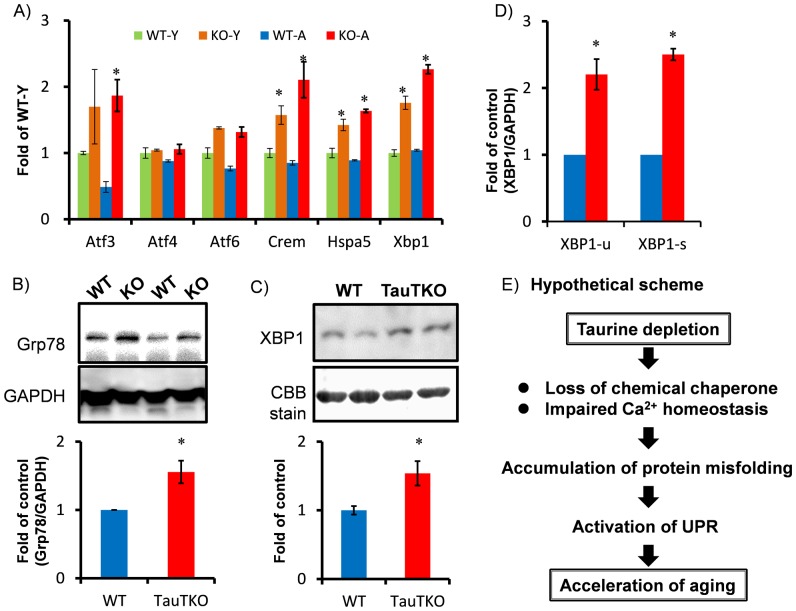
Activation of the unfolded protein response in TauTKO muscle. A) Microarray data show upregulation of UPR-related genes, including ATF3, 6, Crem, Hspa5 (Grp78) and Xbp1, in TauTKO muscle. n = 3. B) Western blot reveals an induction in Grp78 protein expression in old TauTKO muscle. n = 5. C) Western blot reveals an induction in nuclear XBP1 protein expression in old TauTKO muscle. n = 4. D) Real-time PCR analysis for unspliced and spliced XBP1 (XBP1-u and XBP1-s, respectively) mRNA in WT and TauTKO mice show a significant induction in both total and spliced (active form) of XBP1 mRNA. n = 5. *;p<0.05 vs WT. E) Our data hypothesize that taurine depletion in skeletal muscle may lead the activation of UPR due to the accumulation of unfolded proteins, which in turn accelerates tissue aging in TauTKO mice.

Our data suggest that tissue taurine depletion of skeletal muscle accelerates the aging process. To uncover the mechanism involved in acceleration of tissue aging in the TauTKO mouse, transcriptome assays and pathway analysis were carried out on skeletal muscle samples isolated from both young and older TauTKO and WT mice (Table S2,S3). To evaluate the impact of tissue taurine depletion on gene expression, data obtained from mRNA expression patterns were analyzed. Initially, ingenuity pathway analysis (IPA) was performed in a gene set which is more than 1.8 higher than the other three groups. The analysis of biological function revealed significant enrichment of genes involved in cell cycle progression (cell cycle) (ASNS, CDKN2A, E2F2, GDF15, LGALS3, RRAD, TP63), necrosis (Cell death and survival) (APLN, ASNS, ATP2A2, C8orf4, CDKN2A, CX3CL1, CXCL10, E2F2, GDF15, KRT18, LGALS3, MAP3K9, NCAM1, PKP2, PLA2G5, RRAD, TP63, TRIB3, UCHL1) etc. (Fig. 3A, Table S4). Moreover, the upstream analysis in this gene set predicted the activation of putative major upstream factors of the regulated genes, including ATF4, PPARG, CTNNB1, IL6, NFkB, TGFB1 and TNF (Fig. 3B, Table 1). Next, to uncover the changes associated with taurine deletion, IPA was performed in overlapping gene set which is more than 1.8 higher or lower in TauTKO samples than WT samples for each age group. The analysis revealed significant enrichment of genes involved in amino acid metabolism (PRODH, SLC38A2, SLC6A9), protein synthesis (APLP1, EDN1, IGF2BP2, IGHM, SRCIN1, YBX2) and protein folding (DNAJ4, HSPA1A) etc (Fig. 3C, Table S5). Activation of TNF, TGFB1 and inhibition of PPARG and TP53 were predicted by upstream analysis (Table 2). A more detailed search of the gene set involved in the regulation of TauTKO muscle revealed changes in myopathy-related genes (ANKRD1, CSRP3, TIMP1, ACTC1, AHNAK, CILP, TNFRSF12A, MYL4, IFIT3, MYL3) and UPR-related genes (ATF3, CREM, HSPA5, XBP1) (Fig. 4A).

**Table 1:** Upstream analysis by IPA of altered genes of old TauTKO muscle.

Upstream Regulator	Molecule Type	Activationz-score	p-value ofoverlap	Target molecules in dataset
ATF4	transcription regulator	2.366	1.43E-07	ASNS, CDKN2A, GDF15, LGALS3, MTHFD2, PSAT1, TRIB3
PPARG	ligand-dependentnuclear receptor	1.992	3.60E-02	ATP2A2, GDF15, KRT18, TRIB3
HRAS	enzyme	1.98	3.61E-02	ASNS, ATP2A2, CDKN2A, CXCL10, KRT18
IL6	cytokine	1.976	5.16E-02	ATP2A2, CDKN2A, CXCL10, E2F2, KRT18
P38 MAPK	group	1.969	3.05E-03	CDKN2A, CXCL10, MYH3, RRAD, SLC6A12
CTNNB1	transcription regulator	1.969	3.91E-02	CDKN2A, LGALS3, MYH3, NCAM1, TP63
NFkB (complex)	complex	1.925	7.04E-03	ATP2A2, CX3CL1, CXCL10, GDF15, NCAM1, TRIB3
lipopolysaccharide	chemical drug	1.774	2.31E-01	ATP2A2, CX3CL1, CXCL10, LGALS3, PLA2G5, TP63, UCHL1
IL1B	cytokine	1.765	4.09E-02	ATP2A2, CX3CL1, CXCL10, E2F2, GDF15, PLA2G5
JUN	transcription regulator	1.702	1.33E-03	ASNS, CDKN2A, CXCL10, GSTA5, LGALS3, NCAM1
NKX2-3	transcription regulator	1.633	3.86E-05	ASNS, C8orf4, CX3CL1, GDF15, PSAT1, TMEM100
STAT4	transcription regulator	1.546	1.17E-04	ARHGAP15, CXCL10, KRT18, LPIN1, PKP2, RRAD
TNF	cytokine	1.495	2.68E-03	APLN, ATP2A2, CDKN2A, CX3CL1, CXCL10, GDF15, LGALS3,MSLN, NCAM1, PLA2G5, RRAD, ZNF365
TGFB1	growth factor	1.348	1.97E-03	APLN, ASNS, CDKN2A, CX3CL1, CXCL10, GDF15, GSTA5,KRT18, LGALS3, MTHFD2, NCAM1, RRAD, ZNF365

**Table 2:** Upstream analysis by IPA of altered genes of young and old TauTKO muscles.

UpstreamRegulator	Molecule Type	Activationz-score	p-value ofoverlap	Target molecules in dataset
TNF	cytokine	2.438	3.87E-02	BIRC5, CYP27A1, EDN1, GADD45G, IFIT3, LAMC2, MGST1, RFTN1, SERPINB1, TIMP1
TGFB1	growth factor	1.977	1.19E-02	ANKRD1, BIRC5, EDN1, FBLN2, GPRC5B, LAMC2, SLC39A8, TIMP1, TNFRSF12A
TLR3	transmembrane receptor	1.477	7.83E-03	CSRP3, EDN1, IFIT3, TIMP1, TP63
Cg	complex	1.144	1.72E-02	ESM1, IFIT3, RUNX1, SFRP4, TIMP1, TNFRSF12A
TP53	transcription regulator	−1.511	4.16E-02	BIRC5, CYFIP2, FBLN2, GADD45G, HMGN2, HSPA1A/HSPA1B, HSPA1L, MYL4, PRODH, RUNX1, SH3BGRL2, TP63
PPARG	ligand-dependentnuclear receptor	−2.425	2.02E-02	ADIG, BIRC5, Ces1d, FAM57B, MGST1, PRODH

### Enhanced unfolded protein response (UPR) in TauTKO muscle

Since organic osmolytes contribute to protein folding, we tested the hypothesis that tissue taurine depletion leads to the accumulation of misfolded and unfolded proteins in the ER, thereby triggering ER stress. We initially examined the signal pathways involved in UPR to assess potential involvement of ER stress in the pathology of tissue taurine depletion (Fig. 4B). The protein levels of Grp78 were increased in TauTKO muscle compared to age-matched WT controls. Moreover, levels of spliced XBP1 mRNA (XBP1s), which can be translated into the active form of the XBP1 protein, and nuclear levels of XBP1 protein were increased in TauTKO muscles (Fig. 4C).

## Discussion

This study demonstrated that tissue taurine depletion shortens lifespan concomitant with acceleration in tissue aging. In TauTKO skeletal muscle, enhanced expression of p16 is associated with advanced aging. A recent study revealed that p16-positive senescent cells play a key role in the progression of whole body aging, which is a determinant of lifespan. Baker et al. demonstrated that clearance of p16-positive senescent cells by a transgenic technique extends the lifespan of BubR1 progeroid mice [37]. Importantly, removal of p16Ink4a-expressing cells delays the onset of the aging-related, pathologic muscle phenotype in the progeroid mice. Thus, induction of p16 in TauTKO skeletal muscle may contribute to accelerated tissue aging. It is widely accepted that senescent cells enhance the secretion of senescent-sensitive secretary proteins, such as cytokines, chemokines, growth factors, Wnt and matrix metalloproteases [38]. Moreover, these secreted factors accelerate aging of neighbor cells and/or the other tissues, which is associated with aging-related diseases, such as cancer, Alzheimer disease and heart diseases [39]. In this study, the upstream analysis from the microarray data predict that the signaling pathways that are downstream of senescence-associated secretory proteins, such as NF-κB, β-catenin (CTNNB1), p38MAPK, IL6, TGFB1, TNF signal cascades, are activated in old TauTKO muscle, suggesting the involvement of senescence-associated secretory protein-dependent signal cascade. Furthermore, although it has been proposed that tissue taurine depletion causes aging-related disorders in several tissues, including heart and liver, we failed to detect changes in p16 levels in those tissues of aged TauTKO mice.

In this study, we showed that tissue taurine depletion increases the aging process in central nuclei containing myotubes. While we have previously reported the histological change in young TauTKO muscle [24], the myotubes with center nuclei were not found in young WT and TauTKO muscles, indicating this phenotype is due to acceleration of aging in TauTKO mice. Central nuclei in myotubes are a characteristic feature of regenerating muscle. This type of myotube is abundant in dystrophic muscle and muscle after injury. These observations imply that tissue taurine depletion leads to cell injury associated with advanced aging, which in turn increases the regeneration of muscle. Decreasing muscle cell size (sarcopenia) is a critical feature of muscle aging. We have previously reported that skeletal muscle already exhibits a decrease in muscle weight and cross sectional area at an early stage in TauTKO mice. We have also demonstrated that the ultrastructure of skeletal muscle is defective in aged TauTKO mice [24], an indication of aging-associated muscle injury. One mechanism that could contribute to a change in ultrastructure is impaired conjugation of taurine with the wobble base of tRNA^Lys^, a reaction implicated in the development of the mitochondrial disease, myoclonic epilepsy and ragged-red fiber syndrome (MERRF), which leads to the formation of ragged red fibers [36], [40]. However, we failed to detect ragged-red fibers in muscle sections of TauTKO mice stained with Gomori trichrome method. Nonetheless, mitochondrial complex I activity is reduced in aged TauTKO muscle. Importantly, we could not detect the reduction of mitochondrial complex I activity in young TauTKO muscle compared to young WT muscle, indicating that the reduction of mitochondrial complex I activity is due to advancing aging. Since mitochondrial dysfunction is likely an important factor involved in accelerated tissue aging, the decline in complex 1 and associated mitochondria-dependent muscle injury likely contributes to enhance skeletal muscle aging. However, the mechanism by which mitochondrial dysfunction contributes to accelerated aging remains to be determined.

In this study, we demonstrated that ER stress may be increased by tissue taurine depletion in skeletal muscle, suggesting that endogenous taurine contributes to the stabilization of protein folding. Moreover, we found inductions in genes involved in amino acid metabolism (PRODH, SLC38A2, SLC6A9), protein synthesis (APLP1, EDN1, IGF2BP2, IGHM, SRCIN1, YBX2) and protein folding (DNAJ4, HSPA1A) in taurine-depleted muscles, suggesting that tissue taurine depletion affects protein homeostasis. These are consistent with the theory that organic osmolytes act as chemical chaperones. The effect of taurine treatment on ER stress has been previously investigated [41], [42]. Taurine attenuates ER stress induced by homocysteine, although taurine failed to attenuate UPR activation induced by typical ER stress inducers, thapsigargin and tunicamycin [41], [42]. Furthermore, Pan et al. recently reported that taurine suppresses ER stress induced by glutamate in the brain [43]. More recently, Bandyopadhyay *et al*. has demonstrated that different chemical chaperones, such as TMAO, proline and glycerol, differentially influence the folding pathways unique to mutant variants, suggesting that osmolytes play a specific role in protein folding [44]. This rationale may explain the effectiveness of taurine in countering the actions of ER stress inducers.

Cells possess various mechanisms to prevent and eliminate protein misfolding or damage, termed proteostasis (protein homeostasis). However, the proteostasis machinery declines with aging, and damaged proteins accumulate in cells [45], [46]. Importantly, several findings illustrate that loss of proteostasis and accumulation of damaged protein contribute to cellular aging [45], [47]. For instance, the activation of autophagy/lysosomal system by overexpression of FOXO or 4E-BP in muscle prevents the accumulation of damaged proteins with aging and thereby muscle dysfunction in Drosophila [47]. Moreover, knocking out XBP1 or IRE-1 reduces stress resistance and shortens lifespan in nematodes [48], suggesting that UPR is also important for aging. Thus, accumulation of misfolded proteins by tissue taurine depletion may be involved in acceleration of muscular senescence. Moreover, recent findings suggest that aging-dependent muscle disorder may control age-related changes in other tissues and longevity [47], [49]. Demontis and Perrimon have demonstrated that muscle-specific overexpression of FOXO or 4E-BP delays muscle functional decay via enhancing removal of damaged protein and extends lifespan in Drosophila [47]. Stensen et al. have reported that muscle-specific overexpression of AMP-activated kinase (AMPK) extends lifespan in Drosophila, whereas muscle-specific inhibition of AMPK decreases lifespan [49]. Thus, senescent muscle may contribute to aging-related disorders in non-muscle tissues in old TauTKO mice. However, this study could not determine the tissue responsibility for longevity in TauTKO mice. Thus, it would be interesting to investigate the impact of muscle-specific mutation of TauT on the lifespan in the future.

In this study, we found that lifespan is significantly shorter in male TauTKO mice but not in female. Moreover, acceleration of skeletal muscle senescence is less severe in female TauTKO than male. The reason for these gender differences is unclear, so far. Gender effects for lifespan have been reported in some experimental geriatric studies. Treatment of metoformin, an anti-diabetic drug, extends lifespan in female mice but not male [50], [51]. Target disruption of insulin-like growth factor (IGF)-1 receptor or ribosomal protein S6 kinase 1 (S6K1) extends lifespan in female mice, but no effect in male [52], [53]. Moreover, treatment of rapamycin, an inhibitor for mammalian target of rapamycin (mTOR), also extends lifespan in mice, but this effect is larger in female than male [5]. Since these molecules are related to protein synthesis as well as energy metabolism, gender difference in these biological processes may relate to the difference in mechanisms of aging in male and female. Therefore, one possibility explaining the gender differences of the effect of taurine deficiency in aging that the difference of susceptibility against loss of chaperonic capacity by taurine depletion influences to severity of aging. Further studies are necessary to clarify the mechanism underlying gender-specific impact of taurine deficiency.

Since control of calcium handling is also an important action of taurine in skeletal muscle, alteration of calcium homeostasis by taurine depletion may also contribute to impairment of muscle function [54]. Moreover, while calcium homeostasis is impaired during aging, several studies have demonstrated that calcium mishandling may contribute to the progression of skeletal muscle aging [55], [56]. Meanwhile, alteration of ER calcium homeostasis activates signal pathways involved in UPR [57], suggesting that aging-associated alteration of calcium handling may disturb ER function, which in turn contributes to enhanced tissue aging. Therefore, alterations in calcium homeostasis by taurine depletion may also contribute to the accumulation of misfolded proteins and in turn the activation of UPR.

## Conclusions

In conclusion, tissue taurine depletion accelerates muscle aging and shortens lifespan; effects may be related to the enhancement of UPR. This study provides the evidence that taurine acts as an anti-aging molecule and that prevention of chronic taurine depletion may serve as a strategy for delaying skeletal muscle senescence.

## Supporting Information

Figure S1 A) Significant induction of p16INK4A in aged female TauTKO muscle. n = 4 (WT), 3 (TauTKO) B) Significant increase in central nuclei-myotubes is detected in aged female TauTKO muscle. n = 4 (WT), 5 (TauTKO). C) Gomori trichrome stain shows no red-ragged fibers in aged TauTKO muscle. Similar results were obtained from 3 independent experiments.
(PDF)

Table S1 **Top 50 genes increased or decreased in old TauTKO muscles than old WT muscles.**
(PDF)

Table S2 **Top 50 genes increased or decreased in young TauTKO muscles than young WT muscles.**
(PDF)

Table S3 **Biological function identified by IPA of increased or decreased genes of old TauTKO muscle.**
(PDF)

Table S4 **Biological function identified by IPA in overlap genes which are significantly changed in both young and old TauTKO muscle.**
(PDF)

Table S5 (PDF)

Checklist S1 **ARRIVE Checklist.**
(PDF)
