# Supplementary material for: Tissue Depletion of Taurine Accelerates Skeletal Muscle Senescence and Leads to Early Death in Mice
Source: PLoS One. 2014 Sep 17;9(9):e107409. doi: 10.1371/journal.pone.0107409 (PMC4167997; doi:10.1371/journal.pone.0107409)
Supplement: Figure S1 — A) Significant induction of p16INK4A in aged female TauTKO muscle. n = 4 (WT), 3 (TauTKO) B) Significant increase in central nuclei-myotubes is detected in aged female TauTKO muscle. n = 4 (WT), 5 (TauTKO). C) Gomori trichrome stain shows no red-ragged fibers in aged TauTKO muscle. Similar results were obtained from 3 independent experiments. (PDF) [file pone.0107409.s001.pdf]

Suplemental figures

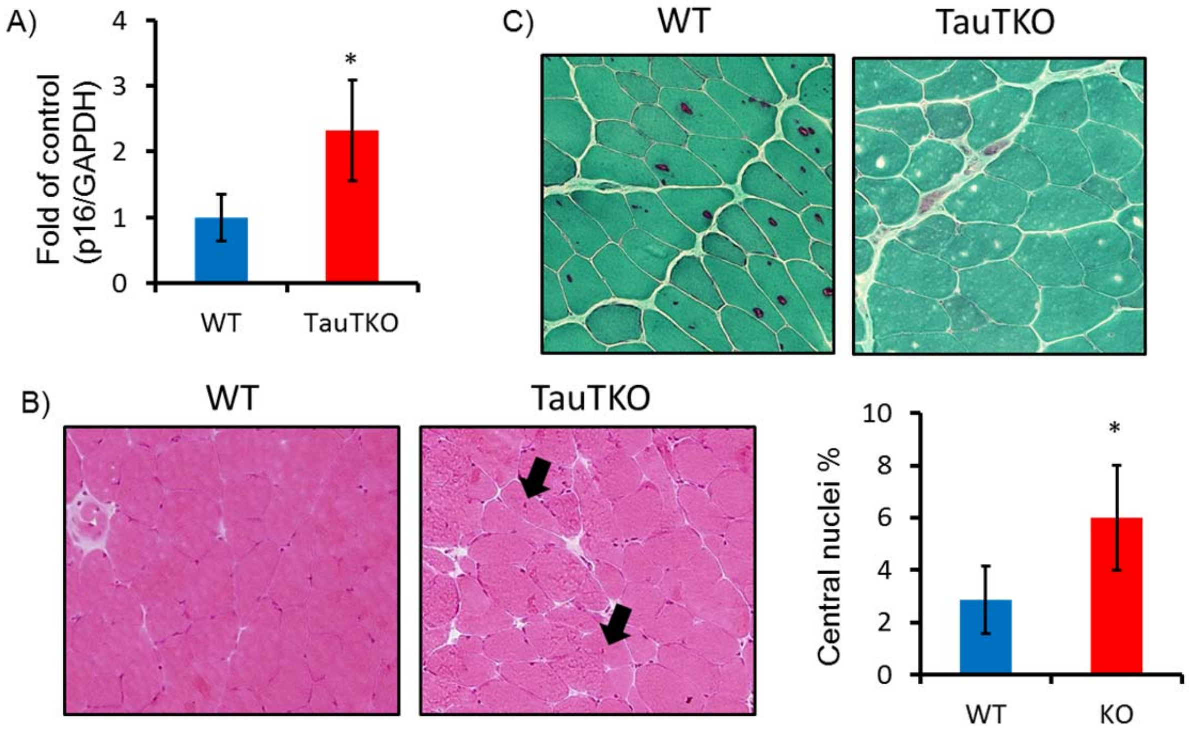

Fig. S1 A) Significant induction of p16INK4A in aged female TauTKO muscle. n=4 (WT), 3 (TauTKO) B) Significant increase in central nuclei-myotubes is detected in aged female TauTKO muscle. n=4 (WT), 5 (TauTKO). C) Gomori trichrome stain shows no red-ragged fibers in aged TauTKO muscle. Similar results were obtained from 3 independent experiments.
