# Supplementary material for: Tissue Depletion of Taurine Accelerates Skeletal Muscle Senescence and Leads to Early Death in Mice
Source: PLoS One. 2014 Sep 17;9(9):e107409. doi: 10.1371/journal.pone.0107409 (PMC4167997; doi:10.1371/journal.pone.0107409)
Supplement: Table S1 — Top 50 genes increased or decreased in old TauTKO muscles than old WT muscles. (PDF) [file pone.0107409.s002.pdf]

Table S1 Top 50 genes increased or decreased in old TauTKO muscles than old WT muscles

Probe Name	Gene Symbol	Gene Name	Fold change (KO-AvsWT-A)
Increase			
A_51_P419117	Rab15	RAB15, member RAS oncogene family	18.503
A_55_P2073552	A930003A15Rik	RIKEN cDNA A930003A15 gene	15.323
A_51_P146044	Ccdc92	coiled-coil domain containing 92	10.369
A_51_P393654	Fam171b	family with sequence similarity 171, member B	9.527
A_51_P241319	Cilp	cartilage intermediate layer protein, nucleotide pyrophosphohydrolase	9.016
A_51_P469951	Srgap3	SLIT-ROBO Rho GTPase activating protein 3	8.390
A_55_P2039320	Zfp365	zinc finger protein 365	7.632
A_52_P21	Ttc9	tetratricopeptide repeat domain 9	7.318
A_51_P324814	Krt18	keratin 18	7.315
A_55_P2092296	Fbxo2	F-box protein 2	6.833
A_55_P2008437	Cdkn2a	cyclin-dependent kinase inhibitor 2A	6.742
A_55_P2052290	Psat1	phosphoserine aminotransferase 1	6.717
A_55_P2044143	Loxl4	lysyl oxidase-like 4	6.629
A_52_P404942	ORF63	open reading frame 63	6.060
A_52_P184149	Mthfd2	methylenetetrahydrofolate dehydrogenase (NAD+ dependent), methenyltetrahydrofolate cyclohydrolase	6.004
A_51_P215374	Slc6a17	solute carrier family 6 (neurotransmitter transporter), member 17	5.607
A_52_P120803	Ankrd1	ankyrin repeat domain 1 (cardiac muscle)	5.496
A_55_P1969306	Ranbp3l	RAN binding protein 3-like	5.467
A_55_P1981366	Lamc2	laminin, gamma 2	5.426
A_55_P2137941	Fxyd2	FXYD domain-containing ion transport regulator 2	5.371
A_51_P261059	Cpne2	copine II	5.296
A_51_P315682	Igf2bp2	insulin-like growth factor 2 mRNA binding protein 2	5.234
A_51_P131408	Tnfrsf12a	tumor necrosis factor receptor superfamily, member 12a	5.166
A_51_P303620	Whrn	whirlin	5.144
A_51_P137604	Fcna	ficolin A	5.069
A_55_P1983448	S100a4	S100 calcium binding protein A4	4.980
A_55_P1982499	Gldn	gliomedin	4.666
A_55_P2050602	Ncam1	neural cell adhesion molecule 1	4.537
A_55_P2078695	Fbxl13	F-box and leucine-rich repeat protein 13	4.344
A_52_P428735	Lrp2bp	Lrp2 binding protein	4.170
A_55_P2068459	Hspa1a	heat shock protein 1A	4.158
A_52_P316933	Sh3bgrl2	SH3 domain binding glutamic acid-rich protein like 2	3.963
A_52_P360330	Mtap1b	microtubule-associated protein 1B	3.867
A_55_P2059864	Igsf1	immunoglobulin superfamily, member 1	3.862
A_52_P49457	Fbxl16	F-box and leucine-rich repeat protein 16	3.806
A_55_P1959748	Asns	asparagine synthetase	3.780
A_55_P2129437	Ppap2c	phosphatidic acid phosphatase type 2C	3.746
A_51_P457196	Sfrp4	secreted frizzled-related protein 4	3.712
A_55_P1984815	Snph	syntaphilin	3.639

A_55_P2121886	Map3k9	mitogen-activated protein kinase kinase kinase 9	3.566
A_52_P452689	Atf3	activating transcription factor 3	3.415
A_55_P2006008	Serpinb1a	serine (or cysteine) peptidase inhibitor, clade B, member 1a	3.357
A_51_P340747	Hspa1l	heat shock protein 1-like	3.299
A_55_P2069818	Mapk10	mitogen-activated protein kinase 10	3.266
A_51_P196844	Osbpl3	oxysterol binding protein-like 3	3.233
A_55_P2010116	Rab27b	RAB27b, member RAS oncogene family	3.221
A_55_P2425938	2810468N07Rik	RIKEN cDNA 2810468N07 gene	3.217
A_51_P214127	Cpa3	carboxypeptidase A3, mast cell	3.200
A_55_P2079619	Rnf43	ring finger protein 43	3.178
A_55_P1985850	Timp1	tissue inhibitor of metalloproteinase 1	3.159
Decrease			
A_55_P1954231	Lrtm2	leucine-rich repeats and transmembrane domains 2	-6.884
A_51_P286748	Frzb	frizzled-related protein	-6.153
A_51_P375969	Ces1d	carboxylesterase 1D	-5.711
A_55_P1999532	Col9a1	collagen, type IX, alpha 1	-5.186
A_52_P257625	Esm1	endothelial cell-specific molecule 1	-4.979
A_51_P260850	Cntnap2	contactin associated protein-like 2	-4.526
A_51_P191726	Efcab6	EF-hand calcium binding domain 6	-4.301
A_55_P2044212	Slc15a5	solute carrier family 15, member 5	-3.797
A_51_P155465	Cuzd1	CUB and zona pellucida-like domains 1	-3.682
A_51_P171200	Golm1	golgi membrane protein 1	-3.622
A_55_P1956534	Dach2	dachshund 2 (Drosophila)	-3.484
A_66_P122699	Cux2	cut-like homeobox 2	-3.477
A_51_P397934	Grin3b	glutamate receptor, ionotropic, NMDA3B	-3.440
A_55_P2020461	Hmgn2	high mobility group nucleosomal binding domain 2	-3.193
A_55_P2094896	Phyhd1	phytanoyl-CoA dioxygenase domain containing 1	-3.192
A_52_P144818	Ppp1r1a	protein phosphatase 1, regulatory (inhibitor) subunit 1A	-3.182
A_55_P2058165	Morn4	MORN repeat containing 4	-3.182
A_55_P2136121	9030617O03Rik	RIKEN cDNA 9030617O03 gene	-3.157
A_52_P354744	Slc2a3	solute carrier family 2 (facilitated glucose transporter), member 3	-3.148
A_55_P2008891	Tmsb15b1	thymosin beta 15b1	-3.120
A_55_P2257670	A030001D16Rik	RIKEN cDNA A030001D16 gene	-3.057
A_51_P369784	Ces1e	carboxylesterase 1E	-3.018
A_55_P2021146	Mchr1	melanin-concentrating hormone receptor 1	-3.014
A_55_P2360356	4933401D09Rik	RIKEN cDNA 4933401D09 gene	-2.928
A_55_P2044710	Asb10	ankyrin repeat and SOCS box-containing 10	-2.889
A_55_P2416494	8430426J06Rik	RIKEN cDNA 8430426J06 gene	-2.865
A_66_P117933	I830012O16Rik	RIKEN cDNA I830012O16 gene	-2.860
A_55_P2005833	Adamts8	a disintegrin-like and metallopeptidase (reprolysin type) with thrombospondin type 1 motif, 8	-2.763
A_52_P266686	Ntsr2	neurotensin receptor 2	-2.746
A_52_P263518	Gng2	guanine nucleotide binding protein (G protein), gamma 2	-2.723
A_55_P2084177	Klhl34	kelch-like 34 (Drosophila)	-2.694
A_51_P401987	Tmem37	transmembrane protein 37	-2.640

A_55_P2172892	1700080G18Rik	RIKEN cDNA 1700080G18 gene	-2.636
A_55_P2057076	Magix	MAGI family member, X-linked	-2.630
A_55_P2008889	Tmsb15b2	thymosin beta 15b2	-2.612
A_52_P368306	Tmem100	transmembrane protein 100	-2.507
A_51_P309854	Kcnn2	potassium intermediate/small conductance calcium-activated channel, subfamily N, member 2	-2.490
A_66_P105350	Cacna2d4	calcium channel, voltage-dependent, alpha 2/delta subunit 4	-2.486
A_51_P289679	Npm2	nucleophosmin/nucleoplasmin 2	-2.466
A_51_P326685	Lrtm1	leucine-rich repeats and transmembrane domains 1	-2.446
A_51_P223404	Plin3	perilipin 3	-2.445
A_51_P109144	Grtp1	GH regulated TBC protein 1	-2.443
A_51_P317191	Eepd1	endonuclease/exonuclease/phosphatase family domain containing 1	-2.428
A_51_P275496	BC026762	cDNA sequence BC026762	-2.427
A_55_P2044547	2810416G20Rik	RIKEN cDNA 2810416G20 gene	-2.397
A_55_P2182716	Adig	adipogenin	-2.353
A_55_P1987805	Rps6ka5	ribosomal protein S6 kinase, polypeptide 5	-2.329
A_52_P589568	Foxo6	forkhead box O6	-2.318
A_55_P1962084	Hist2h2aa1	histone cluster 2, H2aa1	-2.299
A_55_P2107045	Myl4	myosin, light polypeptide 4	-2.287
