# Supplementary material for: Tissue Depletion of Taurine Accelerates Skeletal Muscle Senescence and Leads to Early Death in Mice
Source: PLoS One. 2014 Sep 17;9(9):e107409. doi: 10.1371/journal.pone.0107409 (PMC4167997; doi:10.1371/journal.pone.0107409)
Supplement: Table S2 — Top 50 genes increased or decreased in young TauTKO muscles than young WT muscles. (PDF) [file pone.0107409.s003.pdf]

Table S2 Top 50 genes increased or decreased in young TauTKO muscles than young WT muscles

Probe Name	Gene Symbol	Gene Name	Fold change (KO-AvsWT-A)
Increase			
A_51_P419117	Rab15	RAB15, member RAS oncogene family	5.831
A_55_P2005549	Tnn	tenascin N	5.457
A_51_P250337	Igfn1	immunoglobulin-like and fibronectin type III domain containing 1	4.225
A_55_P2037608	Acp5	acid phosphatase 5, tartrate resistant	4.179
A_51_P131408	Tnfrsf12a	tumor necrosis factor receptor superfamily, member 12a	3.574
A_51_P133684	Csrp3	cysteine and glycine-rich protein 3	3.381
A_55_P2160296	Olfm1	olfactomedin 1	3.326
A_55_P1984815	Snph	syntaphilin	3.321
A_51_P423549	Shbg	sex hormone binding globulin	3.250
A_52_P356093	B3galt2	UDP-Gal:betaGlcNAc beta 1,3-galactosyltransferase, polypeptide 2	3.049
A_51_P469951	Srgap3	SLIT-ROBO Rho GTPase activating protein 3	3.032
A_55_P1954086	Postn	periostin, osteoblast specific factor	2.902
A_55_P1981366	Lamc2	laminin, gamma 2	2.872
A_55_P2092296	Fbxo2	F-box protein 2	2.833
A_51_P430973	Paqr7	progestin and adipoQ receptor family member VII	2.785
A_55_P1961270	Cd72	CD72 antigen	2.696
A_55_P2009604	Gm608	predicted gene 608	2.685
A_55_P1970105	Ltbp2	latent transforming growth factor beta binding protein 2	2.672
A_55_P2137941	Fxyd2	FXYD domain-containing ion transport regulator 2	2.646
A_52_P453785	Cthrc1	collagen triple helix repeat containing 1	2.569
A_51_P241319	Cilp	cartilage intermediate layer protein, nucleotide pyrophosphohydrolase	2.524
A_51_P303620	Whrn	whirlin	2.505
A_55_P2026728	Mylk4	myosin light chain kinase family, member 4	2.469
A_55_P1973447	Ybx2	Y box protein 2	2.458
A_55_P2136906	Vpreb3	pre-B lymphocyte gene 3	2.427
A_55_P1969306	Ranbp3l	RAN binding protein 3-like	2.412
A_51_P457196	Sfrp4	secreted frizzled-related protein 4	2.362
A_51_P205215	Klhl30	kelch-like 30 (Drosophila)	2.352
A_55_P2040951	Actc1	actin, alpha, cardiac muscle 1	2.324
A_55_P2062469	Col12a1	collagen, type XII, alpha 1	2.298
A_52_P167535	BC060267	cDNA sequence BC060267	2.298
A_51_P419017	Pde10a	phosphodiesterase 10A	2.290
A_51_P196844	Osbpl3	oxysterol binding protein-like 3	2.280
A_51_P173709	Gprc5b	G protein-coupled receptor, family C, group 5, member B	2.276
A_55_P2069935	Fxyd2	FXYD domain-containing ion transport regulator 2	2.275
A_55_P2146111	Spink11	serine peptidase inhibitor, Kazal type 11	2.273
A_55_P1979330	Dapp1	dual adaptor for phosphotyrosine and 3-phosphoinositides 1	2.257
A_52_P141338	Acot10	acyl-CoA thioesterase 10	2.247
A_55_P2036083	Gm5797	predicted gene 5797	2.236

A_51_P227392	Rhou	ras homolog gene family, member U	2.207
A_55_P1961335	Ctsk	cathepsin K	2.175
A_55_P2124751	Col16a1	collagen, type XVI, alpha 1	2.170
A_51_P392291	Pdk3	pyruvate dehydrogenase kinase, isoenzyme 3	2.157
A_55_P1962937	Trem2	triggering receptor expressed on myeloid cells 2	2.156
A_51_P244824	Dapp1	dual adaptor for phosphotyrosine and 3-phosphoinositides 1	2.152
A_52_P659312	Spsb4	splA/ryanodine receptor domain and SOCS box containing 4	2.151
A_52_P390944	Chst3	carbohydrate (chondroitin 6/keratan) sulfotransferase 3	2.140
A_51_P458384	Slc38a2	solute carrier family 38, member 2	2.138
A_55_P2006008	Serpinb1a	serine (or cysteine) peptidase inhibitor, clade B, member 1a	2.136
A_55_P1983773	Birc5	baculoviral IAP repeat-containing 5	2.126
Decrase			
A_55_P2152547	Myl3	myosin, light polypeptide 3	-13.405
A_51_P257951	Retnla	resistin like alpha	-5.654
A_55_P1973683	Morn4	MORN repeat containing 4	-4.040
A_55_P2094896	Phyhd1	phytanoyl-CoA dioxygenase domain containing 1	-3.939
A_51_P286748	Frzb	frizzled-related protein	-3.442
A_55_P2044212	Slc15a5	solute carrier family 15, member 5	-3.192
A_52_P18765	Hsbp1l1	heat shock factor binding protein 1-like 1	-3.051
A_52_P175376	Tcfcp2l1	transcription factor CP2-like 1	-2.973
A_55_P2020461	Hmgn2	high mobility group nucleosomal binding domain 2	-2.873
A_55_P2044710	Asb10	ankyrin repeat and SOCS box-containing 10	-2.737
A_51_P375969	Ces1d	carboxylesterase 1D	-2.631
A_55_P2416494	8430426J06Rik	RIKEN cDNA 8430426J06 gene	-2.536
A_66_P123980	2310015K22Rik	RIKEN cDNA 2310015K22 gene	-2.528
A_55_P2257670	A030001D16Rik	RIKEN cDNA A030001D16 gene	-2.507
A_55_P1954231	Lrtm2	leucine-rich repeats and transmembrane domains 2	-2.440
A_51_P191726	Efcab6	EF-hand calcium binding domain 6	-2.438
A_66_P105350	Cacna2d4	calcium channel, voltage-dependent, alpha 2/delta subunit 4	-2.390
A_55_P2175880	Mgst1	microsomal glutathione S-transferase 1	-2.389
A_55_P2070045	Golga7b	golgi autoantigen, golgin subfamily a, 7B	-2.373
A_55_P1999532	Col9a1	collagen, type IX, alpha 1	-2.295
A_51_P260850	Cntnap2	contactin associated protein-like 2	-2.278
A_51_P171200	Golm1	golgi membrane protein 1	-2.252
A_52_P496403	1700001O22Rik	RIKEN cDNA 1700001O22 gene	-2.226
A_55_P2031999	9030617O03Rik	RIKEN cDNA 9030617O03 gene	-2.217
A_51_P401987	Tmem37	transmembrane protein 37	-2.205
A_51_P109369	Fbxo32	F-box protein 32	-2.109
A_55_P2122605	Cbr2	carbonyl reductase 2	-2.100
A_51_P223404	Plin3	perilipin 3	-2.078
A_55_P1961014	Selenbp1	selenium binding protein 1	-2.063
A_55_P2018061	Cd209a	CD209a antigen	-2.056
A_51_P109144	Grtp1	GH regulated TBC protein 1	-2.035
A_55_P2177488	Hist2h2be	histone cluster 2, H2be	-2.023
A_55_P2178510	Mc3r	melanocortin 3 receptor	-1.984
A_52_P311853	Ddit4l	DNA-damage-inducible transcript 4-like	-1.974

A_51_P369784	Ces1e	carboxylesterase 1E	-1.963
A_51_P112734	Slc7a8	solute carrier family 7 (cationic amino acid transporter, y+ system), member 8	-1.952
A_52_P521882	Hddc3	HD domain containing 3	-1.950
A_66_P122699	Cux2	cut-like homeobox 2	-1.949
A_55_P2172892	1700080G18Rik	RIKEN cDNA 1700080G18 gene	-1.942
A_51_P115953	Ctxn3	cortexin 3	-1.926
A_52_P257625	Esm1	endothelial cell-specific molecule 1	-1.925
A_51_P179697	Fam57b	family with sequence similarity 57, member B	-1.887
A_52_P281702	Igfbp5	insulin-like growth factor binding protein 5	-1.874
A_52_P615375	Hist3h2a	histone cluster 3, H2a	-1.872
A_55_P2107155	Rasd2	RASD family, member 2	-1.865
A_51_P215438	Prodh	proline dehydrogenase	-1.858
A_51_P112817	Cyp27a1	cytochrome P450, family 27, subfamily a, polypeptide 1	-1.853
A_52_P126266	Prkab2	protein kinase, AMP-activated, beta 2 non-catalytic subunit	-1.844
A_55_P2057070	Magix	MAGI family member, X-linked	-1.837
A_55_P2002572	Ephx2	epoxide hydrolase 2, cytoplasmic	-1.836
