# Supplementary material for: Tissue Depletion of Taurine Accelerates Skeletal Muscle Senescence and Leads to Early Death in Mice
Source: PLoS One. 2014 Sep 17;9(9):e107409. doi: 10.1371/journal.pone.0107409 (PMC4167997; doi:10.1371/journal.pone.0107409)
Supplement: Table S3 — Biological function identified by IPA of increased or decreased genes of old TauTKO muscle. (PDF) [file pone.0107409.s004.pdf]

Table S3 Biological function identified by IPA of increased or decreased genes of old TauTKO muscle.

Category	Diseases or Functions Annotation	p-Value	Activation z-score	Molecules
Cell Cycle	arrest in G1 phase of breast cancer cell lines	1.12E-04		CDKN2A, GDF15, LGALS3
	cell cycle progression	1.56E-03	1.853	ASNS, CDKN2A, CXCL10, E2F2, KRT18, LGALS3, MSLN, NPM2, RRAD, SPDYA, TP63, ZNF365
	mitosis	8.14E-03	0.747	CDKN2A, CXCL10, E2F2, KRT18, NPM2, ZNF365
Lipid Metabolism	release of prostaglandin D2	2.51E-04		LPIN1, PLA2G5
Cellular Compromise	degranulation of cells	7.84E-04	1.4	C4A/C4B, CX3CL1, LPIN1, NCAM1, TRIB3
Cellular Movement	chemotaxis of microglia	1.07E-03		CX3CL1, CXCL10
	cell movement of granulocytes	1.04E-02	2.219	C4A/C4B, CX3CL1, CXCL10, LGALS3, PLA2G5
Cell Death and Survival	necrosis	3.55E-03	1.178	APLN, ASNS, ATP2A2, C8orf4, CDKN2A, CX3CL1, CXCL10, E2F2, GDF15, KRT18, LGALS3, MAP3K9, NCAM1, PKP2, PLA2G5, RRAD, TP63, TRIB3, UCHL1
	apoptosis	5.32E-03	0.98	APLN, ASNS, C8orf4, CDKN2A, CX3CL1, CXCL10, E2F2, GDF15, KRT18, LGALS3, MAP3K9, NCAM1, PKP2, PLA2G5, RRAD, SPINT2, TP63, TRIB3, UCHL1
