# Supplementary material for: Tissue Depletion of Taurine Accelerates Skeletal Muscle Senescence and Leads to Early Death in Mice
Source: PLoS One. 2014 Sep 17;9(9):e107409. doi: 10.1371/journal.pone.0107409 (PMC4167997; doi:10.1371/journal.pone.0107409)
Supplement: Table S4 — Biological function identified by IPA in overlap genes which are significantly changed in both young and old TauTKO muscle. (PDF) [file pone.0107409.s005.pdf]

Table S4 Biological function identified by IPA in overlap genes which are significantly changed in both young and old TauTKO muscle.

| Category | Diseases or Functions Annotation | p-Value | z-score | Molecules |
|---|---|---|---|---|
| Amino Acid Metabolism | uptake of neutral amino acid | 4.67E-05 | | EDN1, SLC38A2, SLC6A9 |
| | quantity of L-aspartic acid | 1.49E-02 | | PRODH |
| Cellular Growth and Proliferation | formation of connective tissue cells | 4.40E-03 | 0.152 | BIRC5, FRZB, SFRP2, SFRP4 |
| | colony formation of cells | 8.47E-03 | 1.097 | ANKRD1, BIRC5, EDN1, GADD45G, HSPA1A/HSPA1B, LOXL4, RUNX1, SFRP2, TP63 |
| Protein Synthesis | expression of protein | 3.39E-03 | 1.134 | APLP1, HSPA1A/HSPA1B, IGF2BP2, IGHM, SRCIN1, TP63, YBX2 |
| Lipid Metabolism | synthesis of dinoprost | 3.47E-03 | | EDN1, IGHM |
| | quantity of steroid | 1.02E-02 | 1.116 | CES1D, CYP27A1, EPHX2, MCHR1, SHBG, SRGAP3, TIMP1, TNFRSF12A, WNT4 |
| Protein Folding | refolding of protein | 4.08E-03 | | DNAJA4, HSPA1A/HSPA1B |
