# Supplementary material for: Tissue Depletion of Taurine Accelerates Skeletal Muscle Senescence and Leads to Early Death in Mice
Source: PLoS One. 2014 Sep 17;9(9):e107409. doi: 10.1371/journal.pone.0107409 (PMC4167997; doi:10.1371/journal.pone.0107409)
Supplement: Table S5 — (PDF) [file pone.0107409.s006.pdf]

Table S5

gene	Forward primer	Reverse primer
p16INK4a	5’- CCCAACGCCCCGAACT-3’	5’- GCAGAAGAGCTGCTACGTGAA-3’
XBP1-s	5’-GAGTCCGCAGCAGGTC-3’	5’-GTGTCAGAGTCCATGGGA-3’
XBP1-u	5’-ACATCTTCCCATGGACTCTG -3’	5’-TAGGTCCTTCTGGGTAGACC -3’
